# Plant Trait Variation along an Altitudinal Gradient in Mediterranean High Mountain Grasslands: Controlling the Species Turnover Effect

**DOI:** 10.1371/journal.pone.0118876

**Published:** 2015-03-16

**Authors:** David S. Pescador, Francesco de Bello, Fernando Valladares, Adrián Escudero

**Affiliations:** 1 Departamento de Biología y Geología, Universidad Rey Juan Carlos, Madrid, Spain; 2 Institute of Botany, Czech Academy of Sciences, Trebon, Czech Republic; 3 Department of Botany, University of South Bohemia, České Budějovice, Czech Republic; 4 Museo Nacional de Ciencias Naturales, Consejo Superior de Investigaciones Científicas, Madrid, Spain; University of Leipzig, GERMANY

## Abstract

Assessing changes in plant functional traits along gradients is useful for understanding the assembly of communities and their response to global and local environmental drivers. However, these changes may reflect the effects of species composition (i.e. composition turnover), species abundance (i.e. species interaction), and intra-specific trait variability (i.e. species plasticity). In order to determine the relevance of the latter, trait variation can be assessed under minimal effects of composition turnover. Nine sampling sites were established along an altitudinal gradient in a Mediterranean high mountain grassland community with low composition turnover (Madrid, Spain; 1940 m–2419 m). Nine functional traits were also measured for ten individuals of around ten plant species at each site, for a total of eleven species across all sites. The relative importance of different sources of variability (within/between site and intra-/inter-specific functional diversity) and trait variation at species and community level along the considered gradients were explored. We found a weak individual species response to altitude and other environmental variables although in some cases, individuals were smaller and leaves were thicker at higher elevations. This lack of species response was most likely due to greater within- than between-site species variation. At the community level, inter-specific functional diversity was generally greater than the intra-specific component except for traits linked to leaf element content (leaf carbon content, leaf nitrogen content, δ^13^C and δ^15^N). Inter-specific functional diversity decreased with lower altitude for four leaf traits (specific leaf area, leaf dry matter content, δ^13^C and δ^15^N), suggesting trait convergence between species at lower elevations, where water shortage may have a stronger environmental filtering effect than colder temperatures at higher altitudes. Our results suggest that, within a vegetation type encompassing various environmental gradients, both, changes in species abundance and intra-specific trait variability adjust for the community functional response to environmental changes.

## Introduction

Knowing how plant fitness and phenotypic expression change along environmental gradients is essential for understanding the response of plant communities to global change drivers [1]. In this sense, patterns of plant functional trait variation inform not only on community structure but also on population dynamics and the mechanisms affecting ecosystem functioning [2–4]. Shifts in plant functional traits within or between sites and/or species across environmental gradients may also reflect deterministic processes of community organization.

In the case of a well known severity gradient like altitude [5], shifts along elevation have been suggested to explain the functional trait variation at diverse biological levels. For instance, decreases of specific leaf area with elevation within species [6, 7], differences in growth and resource use efficiency traits in populations of vicariant species [8, 9] or functional discrepancies between lowland *versus* highland plant species [10, 11]. In short, different functional responses have been documented along altitudinal gradients, although how to scale up this functional variation to the community level still remains a challenge [4, 12, 13].

One question that remains unanswered is the relative effect of changes in species composition plus abundance (i.e. species turnover) vs. changes in intra-specific trait variability along altitudinal gradients [14]. Previous studies have suggested that intra-specific effects are more important in short environmental gradients, where changes in species composition are small [15]. Nevertheless, in response to shifting environmental conditions, plant communities could modify the intra-specific functional response between sites and/or change species composition (i.e. composition turnover), as well as the abundance of coexisting species. In all cases it would be possible to achieve a new functional configuration as a response to novel conditions. Consequently, when assessing the response of community to novel conditions, we should take into consideration: 1) the dominant trait values along a specific environmental gradient, which can be evaluated using the weighted mean trait value at each site (i.e. community weighted mean—CWM—[14, 16]) and 2) the intra-specific trait variability, which can be considered when assessing the functional diversity (here-after FD, the extend of trait differences in a given community; [17, 18]). Although recent studies are shedding new light on the importance of incorporating within site and intra-specific trait variability in FD calculations [12, 19–22], we are far from reaching an unanimous conclusion [13, 23, 24] because most studies have focused on gradients with high composition turnover (but see [13]). To our knowledge the assessment of intra-specific trait variability and species abundance in systems and vegetation types with almost no species shifts remain almost unexplored.

Bearing these challenges in mind, we focused on a Mediterranean high mountain grassland that occurs far above the timberline and extends along an altitudinal gradient to assess intra- and inter-specific plant functional shifts within and between sites. In this high mountain habitat, environmental is so constraining that only certain well adapted species can thrive (i.e. low composition turnover). Thus, adjustment to varying local conditions along the gradient should mainly rely on intra-specific trait variability between sites or changes in species abundance because the entrance of new species is not feasible and the best trait combination would be selected at each site as a result of a habitat filtering process [25, 26]. What we hypothesize is that either of these two components could track the environmental conditions and modulate the fine-scale functional response at the community level.

In this study, we aimed to answer the following questions: (i) How do individual species respond to shifts in environmental conditions? (ii) How important is within-site and between-site trait diversity for each species and functional trait? iii) What is the relevance of intra-specific *versus* inter-specific FD components and how do they change along the studied altitudinal gradient? and (iv) What is the relative contribution of species turnover and intra-specific trait variability on the response of dominant traits (i.e. CWM) and FD to altitude? We considered information of nine plant functional traits under different sources of variability (within/between sites and intra-/inter-specific) to evaluate the effect of altitude on functional responses in a plant community. Then, based on CWM and FD indices and keeping composition turnover at low level, we assessed the relevance of intra-specific variability *versus* abundance changes for each index.

## Materials and Methods

### Ethics Statement

Permission for field sampling was obtained from the *Dirección General de Medio Ambiente de Madrid*. This study did not involve endangered or protected species.

### Sites and species selection

In the summer of 2011, we selected 9 sites covering the whole altitudinal range of a *Festuca curvifolia* Lag. *ex* Lange grassland community, which is located above the tree-line in the Sierra de Guadarrama (40°46´39´´ to 40°51´8´´ N; 3°49´44´´ to 4°4´59´´W; 1940 m—2419 m a.s.l.; S2 Fig.). This mountain range is located in central Spain, 70 km north of Madrid. The climate is Mediterranean, with average annual temperature and precipitation values of 6.4°C and 1350 mm, respectively (Navacerrada Pass weather station; 40° 47′ N, 4° 0′ W; 1894 m a.s.l.). Due to intense summer drought, summer precipitation only accounts for a small fraction of total annual precipitation (<10%). Stunted Scots pines (*Pinus sylvestris* L.) form a discontinuous timberline between 1900 and 2100 m interspersed in a shrubby matrix formed by *Cytisus oromediterraneus* Rivas Mart. & al. and *Juniperus communis* L. subsp. *alpina* (Suter) Célak. Our pasture-like community extends above this limit, forming high mountain community islands on the highest summits. This community is dominated by several creeping chamaephytes and caespitosous grasses with a biphasic structure in which plants are clumped in a bare ground matrix.

One sampling plot (quadrats 20 x 20 m) was established at each of the 9 sites, trying to minimize variation in slope, orientation and rockiness. In each plot, we identified the set of the most abundant species, representing over 80% total cover. This set was comprised of 10 species at most sites, and a total of 11 species across all sites, showing that species composition between sites was very similar (S1 Table). At each site, 10 random individuals of each species were sampled. Different phylogenetic groups of species were sampled, including a coniferous shrub (*Juniperus communis* subsp. *alpina*), three graminoids (*Agrostis delicatula* Pourr. ex Lapeyr.*; Deschampsia flexuosa* (L.) Trin.*; Festuca curvifolia*), four cushion chamaephytes (*Armeria caespitosa* (Gómez Ortega) Boiss. in DC.; *Jasione crispa* (Pourr.) Samp.; *Minuartia recurva* (All.) Schinz & Thell s.l.; *Silene ciliata* Pourret) and three forbs species (*Pilosella vahlii* (Froel.) F.W. Schultz & Sch. Bip.; *Jurinea humilis* (Desf.) DC.; *Senecio carpetanus* Boiss & Reuter). Among these species, we found elements with a wide range of distribution together with some narrow endemics (*F*. *curvifolia*), Mediterranean high mountain specialists (*J*. *crispa*) and arctic alpine plants (*D*. *flexuosa*).

We measured the altitude and orientation of each site using a GPS (Garmin Colorado-300) and the slope using a clinometer (Silva Clinomaster CM-360-%, LA). Orientation and slope values were used to estimate the insolation coefficient following Gandullo´s method [27, 28]. We sampled species richness in the plot and established five random subplots (2.4 x 2.4 m) to visually estimate species, soil and rock cover. Finally, we randomly collected 5 soil samples (5 cm of diameter, 10 cm depth) in three different microhabitats in each plot: bare ground, vegetated patches dominated by grasses, and shrub areas. Soil samples were sieved (2 mm mesh) and air-dried for 1 month. We then estimated soil organic carbon (SOC) by colorimetry after oxidation with K_2_Cr_2_O_2_ and H_2_SO_4_ [29] and soil total N (NT) on a SKALAR++ San Analyzer (Skalar, Breda, The Netherlands) after digestion in H_2_SO_4_ and Kjedahl´s catalyst. Additionally we have information concerning soil temperature and moisture measured on twelve sites (S2 Fig.; six of which corresponded with the sites where functional traits were sampled) along the whole altitudinal range of a *F*. *curvifolia* grassland community in the Sierra de Guadarrama and during a growth season (June-August). Soil temperature and moisture were measured through TMC20-HD thermistors (Onset, Bourne, USA) and VG400 Soil Moisture Sensor Probes (Vegetronix, Utha, USA) respectively, both attached to HOBO U12 four-channel external data loggers (±2mV ±2.5% of absolute reading; Onset, Bourne, USA).

### Measurement of traits

We measured nine functional traits on the sampled individuals (10 individuals per species and site; S1 Table and S4 Table). Plant size (SI) was estimated using canopy area projection (cm^2^) as a surrogate of accumulated resources, adjusting an ellipsoid shape:

$$SI=\pi \frac{D}{2}\frac{d}{2}$$ (1)

where *D* represents the largest diameter and *d* the smallest perpendicular. Plant height (Hmax, distance from the ground to the highest photosynthetic tissues) is related to competitive vigor and stress tolerance [30]. The leaf economic spectrum was represented by specific leaf area (SLA; ratio of fresh leaf area to dry leaf weight) and leaf dry matter content (LDMC; dry leaf weight divided by fresh saturated leaf weight). SLA is related to potential relative growth or relative photosynthetic rates, while LDMC is related to toughness and resistance to physical hazards and tends to scale to 1/SLA [30]. We weighed 2 fresh well-developed leaves per individual using a microbalance (Mettler Toledo MX5, Columbus, OH; weight uncertainty ±1 μg). Projected surface area was estimated with a digital scanner (Epson Perfection 4870) and Adobe Photoshop CS3 software (Adobe Systems, San Jose, CA). The leaves were then oven-dried at 60°C for 72 hours, and dry mass was measured. We also estimated leaf thickness using a dial thickness gauge (Mitutoyo Co., Aurora, IL, USA). This trait is related to resource acquisition and is often correlated to SLA and LDMC [31]. Finally, leaf carbon content (LCC), Leaf Nitrogen Content (LNC) and carbon and nitrogen isotope ratios of organic material (δ^13^C and δ^15^N) were measured in a PDZ Europa ANCA-GSL elemental analyzer interfaced to a PDZ Europa 20–20 isotope ratio mass spectrometer (Sercon Ltd., Cheshire, UK). These two isotopes are interesting traits, because δ^13^C is linked to water-use efficiency (drought tolerance), and δ^15^N reveals the net effect of multiple processes, such as mycorrizal associations, dynamics in atmospheric/soil N-sources or changes in internal N cycles [32].

### Data analyses

First, we conducted a principal components analysis by using a matrix with each individual and trait value (856 individuals x 9 traits) to determine the overall patterns and relationships among all plant traits, individuals and species.

We then built a series of models to understand the response and variation of each functional trait and species along different environmental gradients. We built linear mixed-effect models for each plant trait, considering altitude as a fixed factor and site as a random factor. Sites were considered a random factor because a certain level of dependence between all the individuals at a site is expected. This approach is conservative because part of the total variation of these plant traits along altitude should be brushed by this random effect. We developed additional mixed-effect models with other fixed factors at the site level, such as insolation, soil properties (i.e. SOC and NT) and soil temperature and moisture (see above for details). In temperature and moisture models we only used a partial data base based on those sites where the temperature and moisture information were available (six sites; see S1 Fig. and S2 Fig.). Our experimental design (10 individuals per species and site) allowed us to survey within-site and between-site variance partitioning fitting a one-way ANOVA for each trait and species, where site was the independent variable [33]. Based on the Sum of Squared decomposition, SS_Total_ = SS_site_ + SS_residuals_, the first term corresponds to total variability; SS_site,_ to between-site variability; and SS_residual,_ to unexplained variability which would be assigned to within-site variability.

At the community level, we estimated two functional metrics. First, we estimated community weighted mean values at each site (CWM, [14, 16]) to explore community shifts in plant traits along altitude. CWM represents the mean trait value of a community considering the relative abundance of each species at a specific site (measured here as species cover). We used two CWM values following Lepš *et al*. approach [14]; using mean trait value measured for each species at a given site (CWM_s_) or using mean value across all sites (CWM_f_):

$$CWM_{s}=\overset{S}{\underset{i=1}{\sum }}p_{i}x_{i\_site}$$ (2a)

$$CWM_{f}=\overset{S}{\underset{i=1}{\sum }}p_{i}x_{i}$$ (2b)

where S is the number of species in a given site, p_i_ is the cover of the i-th species in the site, x_i_plot_ is the trait value of the i-th species for the considered site and x_i_ is the trait value of the i-th species for all sites where the species is found. This approach identifies the causes of changes in CWM across a gradient, which come from three resources of variability: intra-specific trait variability vs changes in species turnover (i.e. species composition-intentionally low here- and abundance) plus the covariance between them [14]. Variation in CWM_s_ results from intra-specific and species turnover changes, while CWM_f_ is only dependent on species turnover, because each individual species has the same average value at all sites. Consequently, the effect of intra-specific trait variability is due to differences between CWM_s_ and CWM_f_. To quantify the relative contribution and test the significance of these two effects, we ran three parallel ANOVAs, on CWM_s_ (i.e. species turnover and intra-specific effects), on CWM_f_ (i.e. species turnover effect) and on their differences (i.e. intra-specific trait variability effect). The total sum of squared (SS) of each ANOVA was decomposed into the variability explained by each individual term according to altitude and an error term (i.e. unexplained variability). We defined a covariation effect on total variability to consider the positive (i.e positive covariation term) or negative (i.e negative covariation term) correlation between species turnover and intra-specific effects [17]. We further carried out a paired t-test to determine if the two CWM were different. Besides we calculated the functional diversity (FD) according to the Rao quadratic entropy metric [34], which performs the sums of all possible trait pairwise dissimilarities between species weighted by relative abundance at each site. Subsequently we divided the functional diversity of each site into intra-specific and inter-specific components according to equation 3 in [17]:

$$\begin{array}{l} \overset{Nind}{\underset{a=1}{\sum }}\overset{Nind}{\underset{b=1}{\sum }}P_{a}P_{b}\frac{d_{ab}^{2}}{2}=\overset{Nsp}{\underset{i=1}{\sum }}\overset{Nsp}{\underset{j=1}{\sum }}p_{i}p_{j}\frac{d_{ij}^{2}}{2}\\ +\overset{Nsp}{\underset{i=1}{\sum }}p_{i}\overset{Nind}{\underset{a_{i}=1}{\sum }}\overset{Nind}{\underset{b_{i}=1}{\sum }}\frac{1}{Nind_{i}}\frac{1}{Nind_{i}}\frac{d_{ab_{i}}^{2}}{2} \end{array}$$ (3)

where the left-hand side of this equation expresses the total diversity, while the right hand of equation shows inter-specific (first row) and intra-specific diversity (second row). The total diversity is represented by the average dissimilarity between each pair of individuals of species considered (*d*_*ab*_) weighted by the relative abundance of these species (*P*_*a*_ and *P*_*b*_). The inter-specific diversity term is estimated by trait dissimilarity between each pair of species *i* and *j* (*d*_*ij*_) considered their relative abundance (*p*_*i*_ and *p*_*j*_). For each species, the intra-specific diversity is depicted by the average dissimilarity between each pair of individuals within species considered (*d*_*abi*_), weighted by the relative abundance of this (*p*_*i*_) and the number of individuals sampled (Nind_i_). The response of each functional metric to altitude (i.e. CWM and FD) was evaluated using linear models. Finally, as for CWM, FD values were computed using site-specific values (FD_s_) or averaged values across all sites (FD_f_) to evaluate the importance of intra-specific variability and/or species turnover effects along altitude [17].

Statistical analyses were carried out using the software R 3.1.0 [35] and the packages *ade4*, *lme4*, *lmerTest*, *FD* and *gplots* and *trait*.*flex*.*anova* function [14] to decompose effect of intra-specific trait variability and/or species turnover on FD response to altitude.

## Results

**Fig 1:**
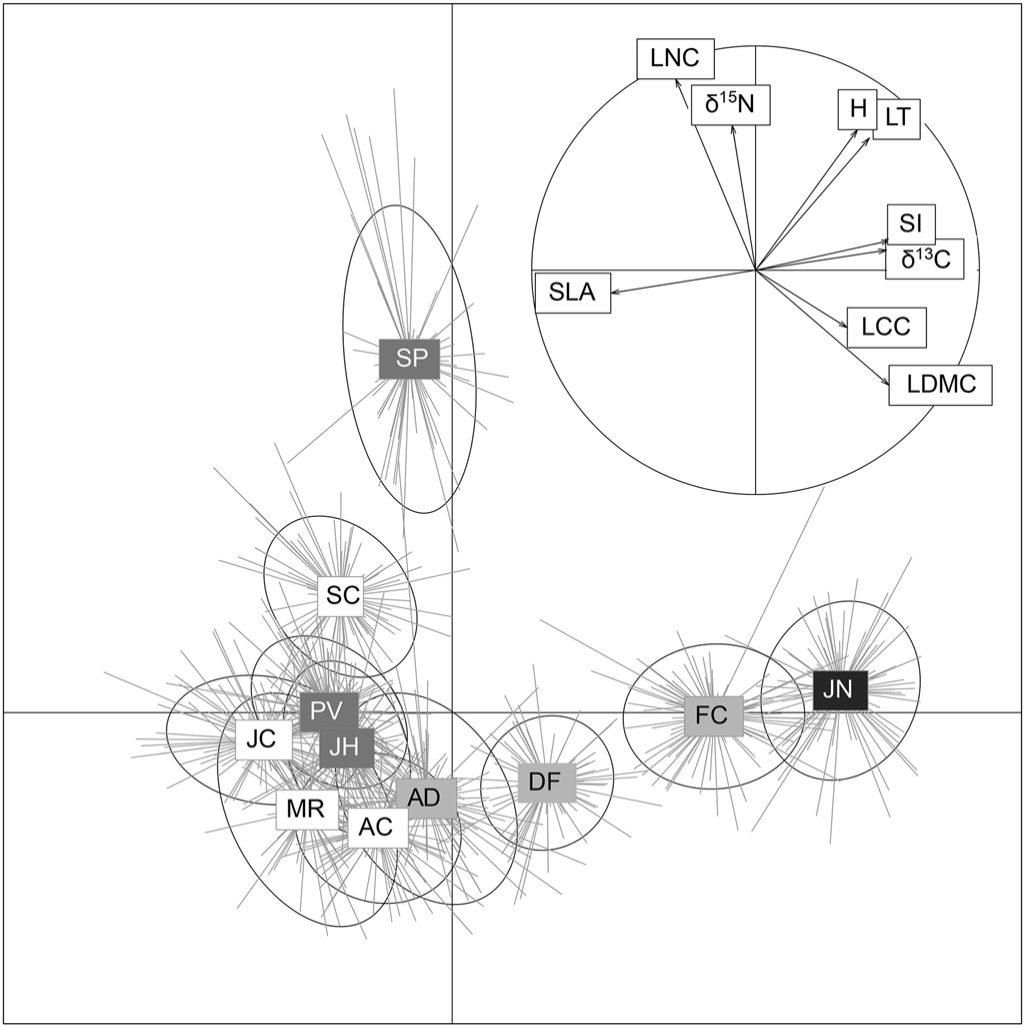
Principal component analysis (PCAs) using the nine traits measured in eleven species for a total of 856 individuals. The variability of individuals of each species and their distributions along trait axes is represented in the main PCA by lines that arise from species mean value and by an ellipse of dispersion. The minor PCA shows the correlation between the nine traits using all data. Species names are included by using acronyms and colour-coded by their growth form: (i) Hemicryptophyte (dark grey): PV (*Pilosella vahlii*), SP (*Senecio carpetanus*), JH (*Jurinea humilis*); (ii) cushion chamaephyte (white): AC (*Armeria caespitosa*), JC (*Jasione crispa*), MR (*Minuartia recurva*), SC (*Silene ciliata*); (iii) caespitosous hemicryptophyte (light grey) FC (*Festuca curvifolia*), DF (*Deschampsia flexuosa*), AD (*Agrostis delicatula*); (iv) shrub (black) JN (*Juniperus communis* subsp. *alpina*). Acronyms for traits: plant size (IS), plant height (H), leaf thickness (LT), specific leaf area (SLA), leaf dry matter content (LDMC), leaf carbon content (LCC), leaf nitrogen content (LNC), carbon and nitrogen isotopes ratios (δ^13^C and δ^15^N, respectively).

The principal components analysis (Fig. 1) showed general patterns of variability as well as relationships between functional traits and species. The first axis was associated with SLA, δ^13^C and individual size, explaining approximately 40% of total variance, whereas the second axis was related to LNC, δ^15^N and height, explaining about 20% of total variance. We found an evident intra-specific variability with a core of 6 species with an equivalent functional traits distribution on the bottom-left quadrant, *F*. *curvifolia* and *J*. *communis* subsp. *alpina* around the positive values of the first axis (high values of individual size and δ^13^C) and *Senecio carpetanus* on the positive values of the second axis (high values of LNC and δ^15^N; Fig. 1, S1 Table).

The correspondence between plant trait values per species and altitude was weak for most traits. However, some species decreased in size with altitude, and others thickened their leaves (S2A Table). Other functional traits showed opposed responses along altitude. Thus, LDMC values increased with altitude in *Minuartia recurva*, and decreased in *Pilosella vahlii*, while the isotopic values of δ^13^C were significantly reduced with the altitude in the case of *Jurinea humilis* whereas it increased in *F*. *curvifolia* (see, S2A Table for more details). In the models where other environmental predictors were used, we found few but idiosyncratic responses (S2C-G Table), for this reason we focused mostly on altitude. For example, high levels of soil organic carbon (SOC) and total N (NT) were positively linked to δ^15^N isotope in the leaves of some species and negatively related to isotopic values of δ^13^C in some species (S2D-E Table). Additionally, temperature increase led to low levels of LCC and LNC in some species.

**Fig 2:**
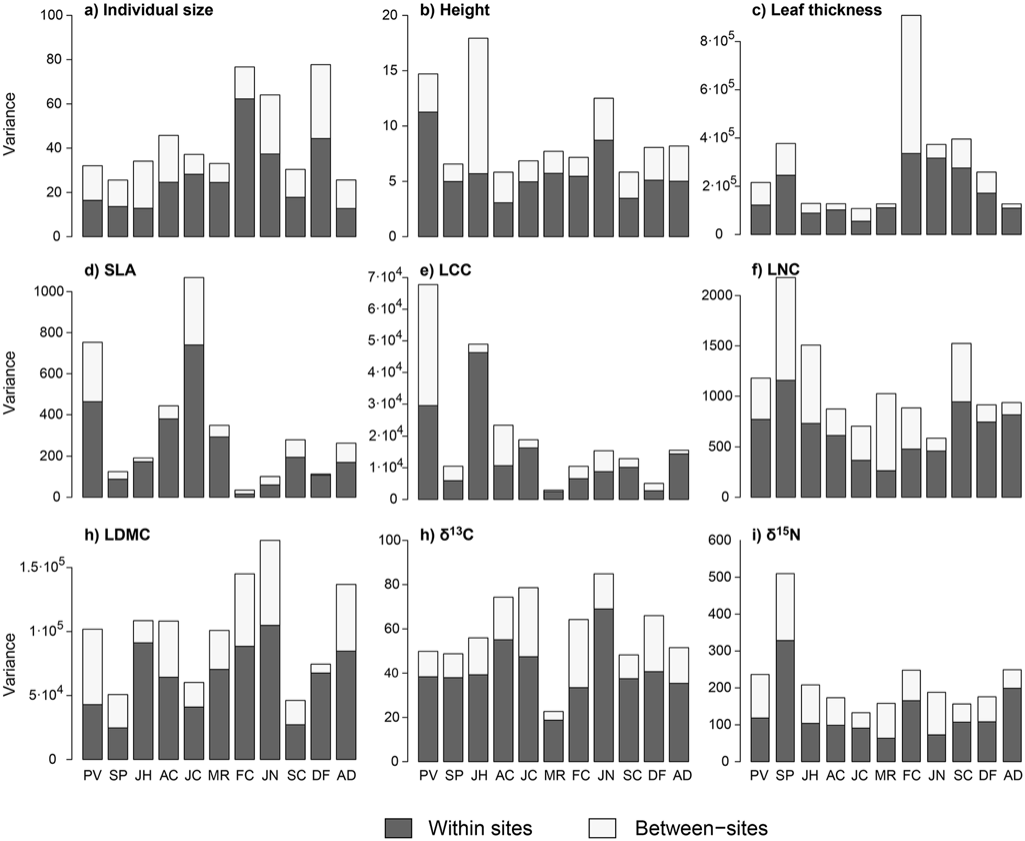
Partition of variance between and within sites for each species and trait (a-i). Decomposition of variance into two levels, within and between sites (grey and white bars respectively), obtained from an analysis of variance model for each of the eleven species and nine traits. Species and trait labels are the same as in Fig. 1.

Intra-specific trait variability partitioning within and between sites were similar for most species and plant functional traits, although slight differences can be observed (Fig. 2). In general, greater variation was found for all species within sites than between sites (on average 65% of the variance corresponded to within-site variation, whereas 35% corresponded to between-site variation).

**Fig 3:**
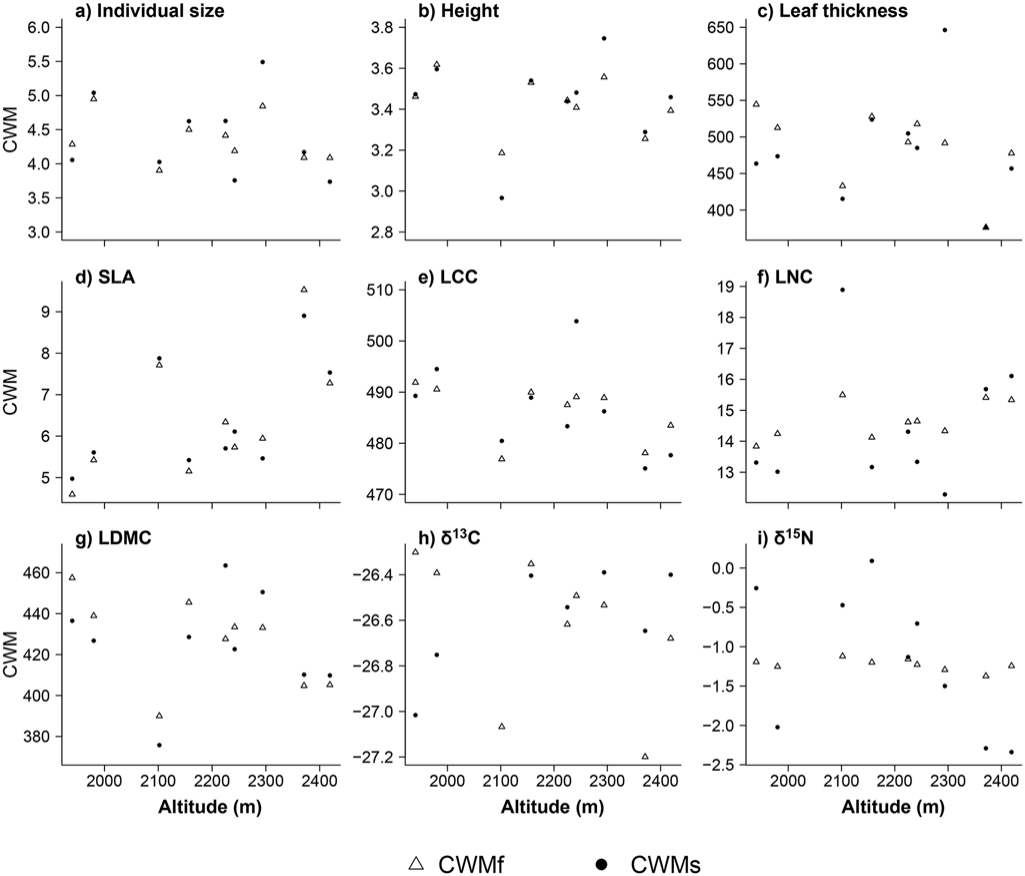
Variation of community weighted means values (CWM) along altitudinal gradient for each functional trait (a-i). Response of two CWM values along altitudinal gradient: using mean trait value measured for each species across all sites (CWM_f_—open triangles) and mean trait value at each site (CWM_s_—black dots). Trait labels are the same as in Fig. 1.

Regardless of whether we regard the mean trait value at each site (CWM_s_) or across all sites (CWM_f_) both values for each community estimate of functional trait performance were quite similar (paired t-test p-value > 0.68 in all cases; Fig. 3). No significant changes in CWM_f_ or CWM_s_ were detected along altitude for any of the functional traits (Fig. 3). The relative contribution of species turnover and/or intra-specific trait variability on CWM response to altitude were very low and generally not relevant, except in the case of intra-specific δ^13^C variability (S3A Table).

**Fig 4:**
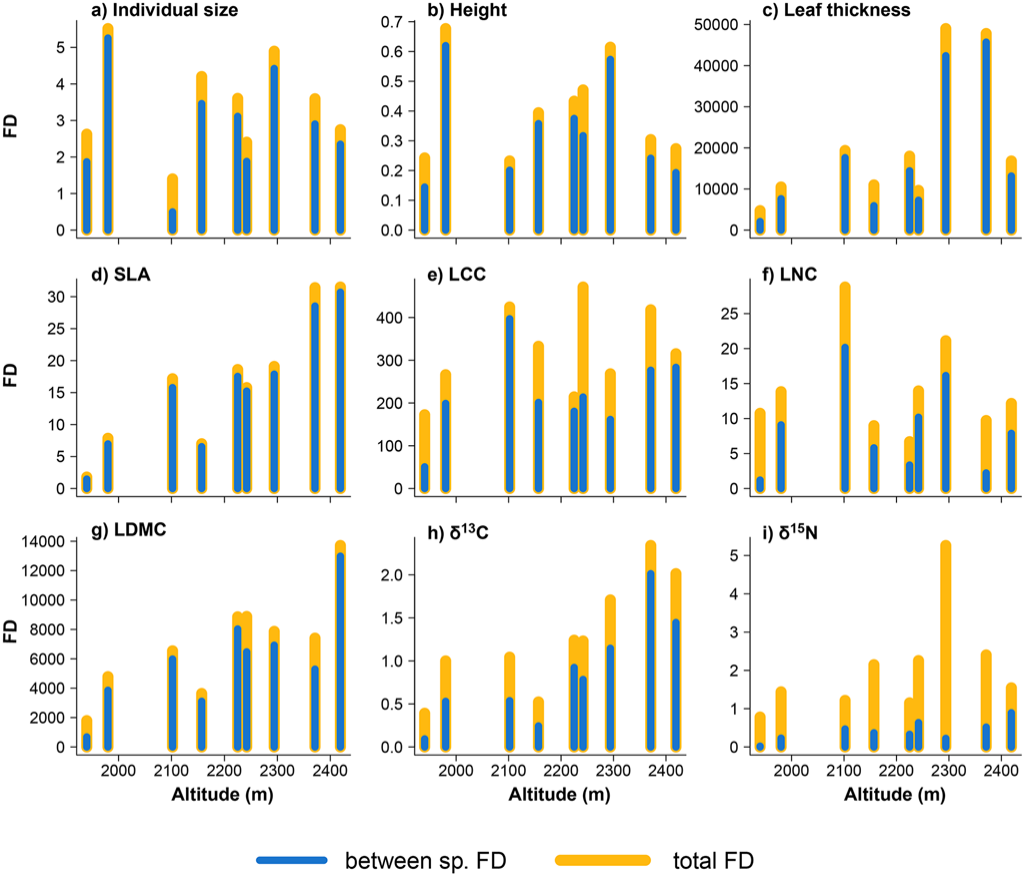
Variation of functional diversity (FD) along altitudinal gradient for each functional trait (a-i). Partition of FD into inter- and intra-specific variability (difference between Total—inter-specific FD) expressed as yellow and blue vertical bars respectively along altitudinal gradient. Trait labels are the same as in Fig. 1.

Overall, the inter-specific FD was generally greater than that of the intra-specific component (Fig. 4). Nevertheless, in some cases, the two components were similar (e.g. LCC), while in others, intra-specific FD was significantly higher, as in δ^15^N and LNC, or δ^13^C. Total and inter-specific FD increased significantly with altitude in the case of SLA (F_1–7_ = 31.92; p < 0.01), LDMC (F_1–7_ = 15.45, p < 0.01), δ^13^C (F_1–7_ = 19.33, p < 0.01) and δ^15^N (only for inter-specific FD; F_1–7_ = 8.8, p = 0.02). When we separated the effect of species turnover and/or intra-specific trait variability on FD response to altitude, we found a significant species turnover effect in the case of SLA (relative contribution of turnover = 65%, p < 0.01). Furthermore, the effect of intra-specific trait variability on FD response to altitude was relevant in δ^13^C (relative contribution of intra-specific variability = 18%, p = 0.01) and in δ^15^N (relative contribution of intra-specific variability = 29.8%, p = 0.01). See S3B Table for more details.

## Discussion

Our study highlights the importance of decomposing trait variability at different levels when assessing plant trait response to abiotic factors even in communities with few changes in identity and in species composition. Surprisingly, within-site trait variability at the individual species level was greater than between-site trait variability. Altitude and other environmental variables, had a weak effect on this variation, even though the species were located along a 500 m altitudinal gradient, which implies a sharp variation in temperature and moisture during the growth season (decreased up to 2°C and increased around 40% respectively; see S1 Fig. for details). At the community level, the inter-specific FD component was generally higher than the intra-specific component and correlated with altitude for some traits. Overall, our results suggest that the functional response of a plant community with almost no species turnover is achieved by modifying the abundance of species in the community rather than by varying considerably the specific functional trait composition between sites.

### Individual species responses along gradients

Although specific trait shifts along environmental gradients have been described as a general pattern [16, 36], we did not detect significant changes in trait values along the different environmental gradients considered for most species and traits. Nevertheless, a trend of decreasing individual size and increasing leaf thickness with increasing altitude was observed in some species. This trend was also observed along some parallel edaphic gradients (i.e. soil organic carbon and total N) in the case of δ^13^C and δ^15^N traits.

Decreasing individual size with altitude is concordant with the growth-limitation hypothesis [37]. In tree and shrub life-forms, low temperatures limit the formation of new cells and tissues, increasing the storage of non-structural carbohydrate compounds mainly in root tissues. This effect was observed in *Juniperus* sp. [38], but to our knowledge, it has not been observed in alpine herbs or creeping chamaephytes. Increasing leaf thickness with elevation has also been described [39]. Recent studies have related this increase to UV-B radiation and the need to protect underlining photosynthetic systems [40]. Complementary mechanisms may inhibit cell expansion in drier environments [41], which would be compatible in our system where drought is more pronounced at low altitudes (S1 Fig.). Furthermore, some authors [42] have suggested a strong link between plant δ^15^N and N of soil in alpine ecosystems. Plants are integrators of δ^15^N of soil N sources in their N nutrition [43, 44], which lead to different foliar δ^15^N signatures in accordance with the dominant physiological and biogeochemical process in the N cycle [45]. This would explain our findings of a significant positive relationship between δ^15^N values and levels of soil total N for *P*. *vahlii*, *J*. *humilis*, *F*. *curvifolia* and *D*. *flexuosa*.

Although we postulated a similar pattern for all individual species in our community in response to altitudinal gradient, our findings were far from clear. The response at broad spatio-temporal scales of each species depends on its biogeography and selective forces, while the species response at finer scales depends on its ability to face micro-environmental heterogeneity and/or biotic interactions. There is usually not a sole conclusion in the study of these patterns [12, 46], as species can have multiple or even opposite responses to one or more environmental gradients. Thus different functional traits may respond to different factors (e.g. δ^13^C is related to aridity gradient [47] while SLA respond to temperature more intensely [48]). In our case, and denoting a striking difference to other mountain ecosystems where moisture availability is not a major limiting factor for plants, we found two opposed severity gradients operating along the altitudinal gradient (drought stress which decreased with altitude and temperature stress which increased with altitude; S1 Fig.). The combined effect of these two opposed forces could explain our results since species net responses can become neutral. As an alternative but also complementary possibility, the indirect effect of temperature and moisture on SOC and NT levels may be not ruled out [49–51] and leads to different responses for each species in the communities at the different sites (S2D-E Table; [52]).

### Within- and between-site variance partitioning

Between-site variability was expected to be the main source of trait variability at the species level, because the habitat filtering and environmental differences between sites would select the best trait combination at each site. However, the highest level of trait-variance for individual species occurred within sites. Greater values of within-site variability were also found in a study conducted in a very heterogeneous temperate mountain ecosystem [12]. These authors expected the opposite results, because they used very small fragments with high genetic similarity in homogeneous micro-environments. Genetic variability and phenotypic plasticity represent two factors that determine intra-specific variability in different environments [15, 53]. Although these two factors are not disentangled using our observational study, parallel studies carried out in this mountain range revealed high within-site genetic variability in two of our study species (*S*. *ciliata* [54] and *A*. *caespitosa* [55]). This relatively high genetic variability could explain our results. Furthermore, soil heterogeneity and micro-topography at fine spatial scales have proven to be as good predictors of plant performance as altitude in alpine ecosystems [56]. Our results suggest that some species and traits could be affected by local heterogeneity rather than by environmental gradients between sites within their realized niche.

### Patterns at the community level

The great intra-specific within-site variability obtained can help us to understand the low variability observed in the CWM along altitude at the community level. Previous studies have found that CWM showed consistent variation under different gradients [57, 58]. Such shifts in CWM could be mainly related to composition changes, in terms of species identity, along gradients, unlike our case where species composition remained similar (73% of species were found in 89% of the sites) which results in low beta diversity (i.e. 1.15; defined as the ratio of the total species diversity in all sites by the mean species diversity at the site level; [59]). Therefore, the low variability in trait values between sites and the lack of shifts in CWM suggest that the species studied are filtered out by the abiotic conditions occurring along altitudinal gradient and confirm that abiotic filters are operating in the community at larger scales [58].

The partition of total functional diversity showed the extent of inter-specific FD was higher than intra-specific FD for 5 out of 9 plant traits (20% intra-specific variability). Although intra-specific trait variability has recently gained strength in the theory of coexistence [60], most studies have found that inter-specific variability is as high and similar as in our study (review Table 1 in [13]). This is surprising, as we expected a higher prevalence of intra-specific variability due to small species turnover [15]. Among the traits with an exceptionally high proportion of intra-specific FD were those associated with nutritional content or some metabolic pathways (δ^15^N and leaf N with 76% and 45% intra-specific FD, respectively), as reported in other studies [13, 19, 46]. These results could be at least partially due to the great effect of soil nutrient availability (which is very variable at small spatial scales in stressful ecosystems) on these traits and/or to genetic differences within species [13, 30].

The relationship between total and/or inter-specific FD and altitude showed a significant shift in 4 out of 9 plant traits. Namely, the highest altitude sites were functionally more diverse for SLA, LDMC, δ^13^C and δ^15^N (only for inter-specific FD). An analogous study conducted in the Alps detected the opposite pattern for SLA and LDMC, finding greater diversity in these traits at low elevations [58]. In contrast to temperate mountains such as the Alps, Mediterranean high mountains are characterized by the presence of two opposing gradients: cold temperature *versus* summer drought. Under this opposite forces, conditions on summits could be actually less stressful due to greater soil water content (S1 Fig.); so coexisting species must maximize their functional diversity with different strategies to coexist and overcome resource competition [57]. In contrast, water limitations at low altitudes could lead to greater abiotic stress [61, 62], which would result in trait convergence and therefore a decrease of functional diversity for these leaf traits [63, 64], but see [65] for a discussion on how competitive exclusion associated with favourable conditions can sometimes drive convergent trait patterns.

On the other hand, the role of intra-specific variability is evident in the study of patterns along different gradients and has helped us to understand the response of community to environmental shifts [22]. For instance, the relative contribution of intra-specific trait variability on CWM and FD response to altitude was significant for δ^13^C and δ^15^N (S3 Table). Although we selected a community with fewer changes in species composition than in most community ecology studies we found evidence of species turnover effect on FD response to altitude for SLA trait (likely due to changes in species relative abundance). This suggests that species turnover can mask the underlying drivers and mechanisms governing community assembly, which should be taken into account in future community studies based on functional diversity and trait patterns. At the same time, FD patterns can be interpreted as a consequence of a strong abiotic filter when environmental conditions become harsher. Nevertheless most of our species showed a weak functional response along altitude with higher within-site variability in most traits and species. This implies that changes in the dominance and abundance in the community and not necessarily shifts in the specific trait response variation between sites (high importance of the within variation in a species) can help to adjust the community functional response to changing environmental conditions when the set of species available in a given species pool of a vegetation type is constrained.

## Supporting Information

S1 Fig Mean soil temperature and moisture in the study area. (PDF)

S2 Fig Location of area studied in the Sierra de Guadarrama (Spain). (PDF)

S1 Table Functional traits and species characterization. (PDF)

S2 Table Response of individual functional traits along several environmental variables: A) altitude, B) squared altitude, C) insolation coefficient, D) soil organic carbon (SOC), E) soil total nitrogen (NT), F) soil temperature and G) soil moisture. (PDF)

S3 Table Species turnover and intra-specific trait variability relative contribution (in %) on A) CWM and B) FD response to altitude. (PDF)

S4 Table Functional trait values for 856 individuals of eleven species measured in nine sampling sites. Acronyms for the traits: leaf thickness (LT), specific leaf area (SLA), leaf dry matter content (LDMC), leaf carbon content (LCC), leaf nitrogen content (LNC), carbon and nitrogen isotopes ratios (δ^13^C and δ^15^N respectably).
(XLSX)
