# Supplementary material for: Plant Trait Variation along an Altitudinal Gradient in Mediterranean High Mountain Grasslands: Controlling the Species Turnover Effect
Source: PLoS One. 2015 Mar 16;10(3):e0118876. doi: 10.1371/journal.pone.0118876 (PMC4361585; doi:10.1371/journal.pone.0118876)

S1 Figure. Mean soil temperature and moisture in the study area. Changes in soil Temperature (ºC; full lines) and Moisture (%; dashed lines) during a growth season (June-August), considering mean region values (upper panel) and mean values at different sites along the altitudinal gradient in the study region (lower panel). These climatic variables were estimated at the following sites: 1 (Porrones, X = 420744, Y = 4512188); 2 (Collado de las Vacas, X = 419169, Y = 4513361); 3 (Loma Cabezas, X = 419960, Y = 4514311); **4 (Collado de la Tirobarra, X = 408746, Y = 4518015)**; **5 (Najarra, X = 430197, Y = 451880)**; 6 (Nevero M, X = 428802, Y = 4537552); 7 (Nevero P, X = 428759, Y = 4537426); **8 (Bola Oeste, X = 417587, Y = 4515896)**; 9 (H. Menor, X = 418456, Y = 4521166); **10 (H. Mayor, X = 418843, Y = 4522259)**; **11 (C. H. Mayor, X = 421276, Y = 4516840)**; **12 (Peñalara, X = 419443, Y = 4522832).** Dots with green rings in the figure and previous bold names denote those sites where traits were also measured.

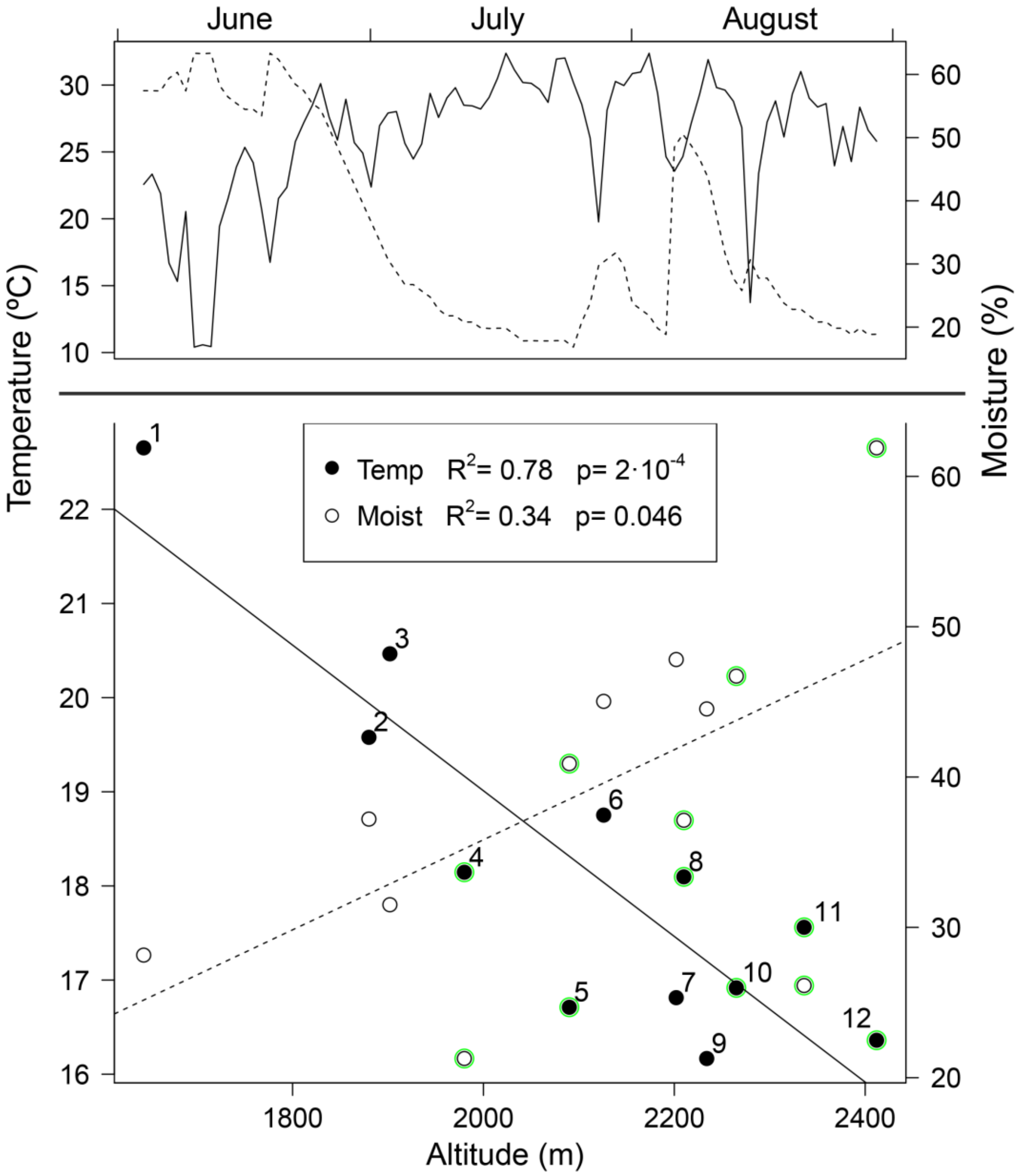

Supplement: S1 Fig — (PDF) [file pone.0118876.s001.pdf]
