# Supplementary material for: Plant Trait Variation along an Altitudinal Gradient in Mediterranean High Mountain Grasslands: Controlling the Species Turnover Effect
Source: PLoS One. 2015 Mar 16;10(3):e0118876. doi: 10.1371/journal.pone.0118876 (PMC4361585; doi:10.1371/journal.pone.0118876)
Supplement: S2 Fig — (PDF) [file pone.0118876.s002.pdf]

**S2 Figure. Location of area studied in the Sierra de Guadarrama (Spain)**. Distribution and altitude (m a.s.l.) of sites where functional traits (green rings) and temperature-moisture information (white dots) were collected.

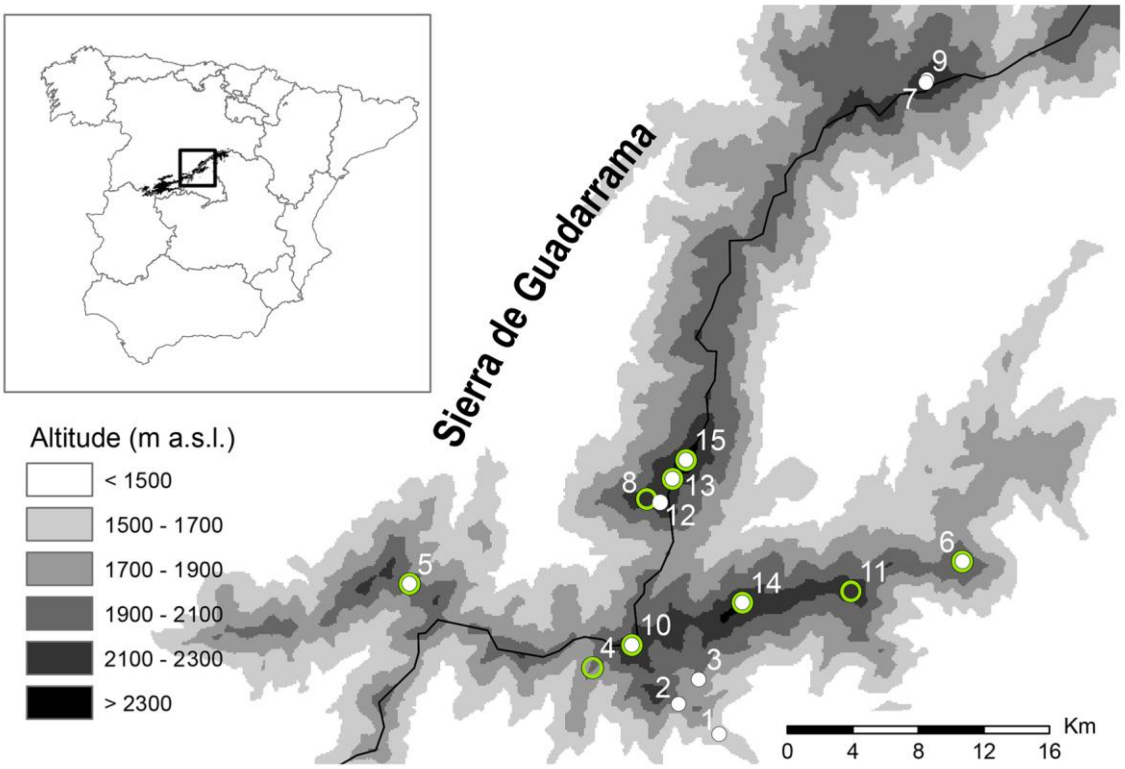


| | | | | | | | |
|---|---|---|---|---|---|---|---|
| 1 | Porrones (1644 m) | 2 | Collado de las vacas (1880 m) | 3 | Loma Cabezas (1902 m) | 4 | Navacerrada (1940 m) |
| 5 | Collado Tirobarra (1980 m) | 6 | La Najarra (2102) | 7 | Nevero M (2126 m) | 8 | Citores (2157 m) |
| 9 | Nevero P (2202 m) | 10 | Bola Oeste (2210 m) | 11 | Asomate-Hoyos (2225 m) | 12 | H. Menor (2234 m) |
| 13 | H. Mayor (2264 m) | 14 | C. H. Mayor (2371 m) | 15 | Peñalara (2419 m) | | |
