# Supplementary material for: Plant Trait Variation along an Altitudinal Gradient in Mediterranean High Mountain Grasslands: Controlling the Species Turnover Effect
Source: PLoS One. 2015 Mar 16;10(3):e0118876. doi: 10.1371/journal.pone.0118876 (PMC4361585; doi:10.1371/journal.pone.0118876)
Supplement: S1 Table — (PDF) [file pone.0118876.s003.pdf]

S1 Table. Functional traits and species characterization. Means and SD (standard deviation) values of the nine functional traits of each of the eleven species sampled. The following species characteristics are shown: label, family, growth form (**HM cae**. - caespitosous hemicryptophyte; **Cs** -cushion chamaephyte; **Sh** - shrub; **Hm forb** – hemicryptophyte) and number of the sites where the species was sampled. Acronyms for the traits: leaf thickness (LT), Specific leaf area (SLA), leaf dry matter content (LDMC), leaf carbon content (LCC), leaf nitrogen content (LNC), carbon and nitrogen isotopes ratios ($\delta^{13}C$ and $\delta^{15}N$ respectably).

Species	Label	Family	Growth form	Sites	Individual size (cm^2)		Height (mm)		LT (µm)		SLA ($mm^2 \cdot mg^{-1}$)		LDMC ($mg \cdot g^{-1}$)		LCC ($mg \cdot g^{-1}$)		$\delta^{13}C$ (‰)		LNC ($mg \cdot g^{-1}$)		$\delta^{15}N$ (‰)	
					mean	sd	mean	sd	mean	sd	mean	sd	mean	sd	mean	sd	mean	sd	mean	sd	mean	sd
Agrostis delicatula	AD	Poaceae	Hm cae.	7	23.45	14.96	20.48	6.79	209.1	42.9	11.43	1.95	397.3	44.5	449.5	15	-28.06	0.86	13.67	3.68	-0.96	1.90
Armeria caespitosa	AC	Plumbaginaceae	Cs	9	9.48	6.66	16.19	4.31	232.3	37.8	15.07	2.23	350.7	34.9	499.6	16.2	-29.38	0.91	14.18	3.13	-1.09	1.40
Deschampsia flexuosa	DF	Poaceae	Hm cae.	8	149.35	200.81	27.57	9.39	302.2	57.2	7.70	1.20	396.9	30.7	459	8	-27.30	0.91	12.04	3.40	-1.67	1.49
Festuca curvifolia	FC	Poaceae	Hm cae.	9	93.74	96.93	32.40	9.75	559.3	101	4.29	0.63	463.5	40.4	492.7	10.9	-26.24	0.85	13.76	3.15	-1.15	1.67
Jasione crispa	JC	Campanulaceae	Cs	8	24.80	18.78	14.99	4.48	188	36.9	18.36	3.68	241.7	27.6	456	15.5	-28.86	1.00	19.76	2.98	-1.20	1.30
Juniperus communis subsp. *alpina*	JN	Cupressaceae	Sh	8	$1.1 \cdot 10^5$	$1.36 \cdot 10^5$	305.05	491.20	417.9	68.7	6.47	1.13	434.4	46.6	502.3	14	-25.70	1.04	12.25	2.73	-2.04	1.54
Jurinea humilis	JH	Asteraceae	Hm forb	9	28.66	18.45	6.96	3.31	262.3	38	10.11	1.47	266.3	34.9	478.5	23.5	-29.26	0.79	22.36	4.11	-1.52	1.53
Minuartia recurva	MR	Caryophyllaceae	Cs	6	31.27	28.44	16.71	6.95	111.2	53.1	17.16	2.78	340.2	47.4	454.1	8.1	-28.67	0.71	17.76	4.77	-1.56	1.87
Pilosella vahlii	PV	Asteraceae	Hm forb	9	15.43	9.62	21.98	8.88	278.1	49.2	16.73	2.91	207.7	33.8	459.5	27.6	-27.55	0.75	20.41	3.64	-2.54	1.63
Senecio carpetanus	SP	Asteraceae	Hm forb	6	35.86	32.01	313.85	89.59	486.7	79.9	10.29	1.45	189.6	29.3	453.9	13.3	-28.86	0.91	35.26	6.08	0.66	2.94
Silene ciliata	SC	Caryophyllaceae	Cs	8	38.81	26.81	19.58	5.13	353.1	70.8	14.06	1.88	207	24.2	440.9	12.8	-27.73	0.78	22.71	4.39	0.30	1.41
