# Supplementary material for: Plant Trait Variation along an Altitudinal Gradient in Mediterranean High Mountain Grasslands: Controlling the Species Turnover Effect
Source: PLoS One. 2015 Mar 16;10(3):e0118876. doi: 10.1371/journal.pone.0118876 (PMC4361585; doi:10.1371/journal.pone.0118876)
Supplement: S2 Table — (PDF) [file pone.0118876.s004.pdf]

S2 Table. Response of individual functional traits along several environmental variables. Summary of linear mixed-effect models scores for each individual plant trait value and species, considering site as random factor and **A)** altitude, **B)** squared altitude, **C)** insolation coefficient, **D)** soil organic carbon (SOC), **E)** soil total nitrogen (NT), **F)** soil temperature and **G)** soil moisture as fixed factors in each model considered. Significant values ($p < 0.05$) are marked in bold.

A)

**lmer(trait$_i$~Altitude+(1|pob)**	Individual size (cm^2)	Height (mm)	LT (µm)	SLA ($mm^2\cdot mg^{-1}$)	LDMC ($mg\cdot g^{-1}$)	LCC ($mg\cdot g^{-1}$)	$\delta^{13}C$ (‰)	LNC ($mg\cdot g^{-1}$)	$\delta^{15}N$ (‰)
	Estimate	Estimate	Estimate	Estimate	Estimate	Estimate	Estimate	Estimate	Estimate
Agrostis delicatula	**$-2.33\cdot10^{-3}$**	$-3.35\cdot10^{-4}$	$-7.90\cdot10^{-3}$	$5.30\cdot10^{-4}$	$7.83\cdot10^{-2}$	$-1.24\cdot10^{-2}$	$-1.25\cdot10^{-3}$	$2.51\cdot10^{-3}$	$-3.36\cdot10^{-3}$
Armeria caespitosa	$-1.44\cdot10^{-4}$	$2.39\cdot10^{-4}$	$-8.19\cdot10^{-3}$	$1.84\cdot10^{-3}$	$-5.76\cdot10^{-2}$	$3.04\cdot10^{-2}$	$1.57\cdot10^{-3}$	$1.42\cdot10^{-3}$	$-2.41\cdot10^{-3}$
Deschampsia flexuosa	$-8.23\cdot10^{-4}$	$5.95\cdot10^{-4}$	**0.17**	$-1.35\cdot10^{-3}$	$2.87\cdot10^{-3}$	$1.98\cdot10^{-2}$	$-2.40\cdot10^{-3}$	$4.99\cdot10^{-3}$	$-3.59\cdot10^{-3}$
Festuca curvifolia	$1.77\cdot10^{-4}$	$2.71\cdot10^{-4}$	0.22	$-1.58\cdot10^{-3}$	$9.73\cdot10^{-2}$	$-1.42\cdot10^{-2}$	**$3.33\cdot10^{-3}$**	$1.40\cdot10^{-3}$	$-3.04\cdot10^{-3}$
Jasione crispa	$-1.44\cdot10^{-3}$	$-1.15\cdot10^{-4}$	$-1.44\cdot10^{-2}$	$2.02\cdot10^{-4}$	$1.68\cdot10^{-2}$	$8.16\cdot10^{-3}$	$1.74\cdot10^{-3}$	$-7.25\cdot10^{-3}$	$6.56\cdot10^{-4}$
Juniperus communis subsp. *alpina*	**$-2.59\cdot10^{-3}$**	$-9.57\cdot10^{-4}$	$-1.49\cdot10^{-2}$	$2.19\cdot10^{-3}$	$-7.02\cdot10^{-2}$	$-3.11\cdot10^{-2}$	$2.56\cdot10^{-4}$	$1.06\cdot10^{-3}$	$-4.22\cdot10^{-3}$
Jurinea humilis	$-7.91\cdot10^{-4}$	$9.95\cdot10^{-5}$	**$9.97\cdot10^{-2}$**	$-9.90\cdot10^{-4}$	$1.95\cdot10^{-2}$	$1.69\cdot10^{-2}$	**$-2.31\cdot10^{-3}$**	$7.19\cdot10^{-3}$	$-1.92\cdot10^{-3}$
Minuartia recurva	$1.46\cdot10^{-3}$	$1.33\cdot10^{-3}$	-0.12	$-7.72\cdot10^{-3}$	**0.21**	$5.88\cdot10^{-3}$	$-8.00\cdot10^{-6}$	$-3.07\cdot10^{-2}$	$-1.05\cdot10^{-2}$
Pilosella vahlii	$-1.52\cdot10^{-3}$	$2.41\cdot10^{-4}$	**0.17**	**$8.34\cdot10^{-3}$**	**-0.14**	$1.54\cdot10^{-2}$	$3.61\cdot10^{-5}$	$-1.59\cdot10^{-3}$	**$-5.77\cdot10^{-3}$**
Senecio carpetanus	**$-2.58\cdot10^{-3}$**	$1.76\cdot10^{-4}$	0.20	$1.57\cdot10^{-3}$	$-5.87\cdot10^{-2}$	$-2.29\cdot10^{-2}$	$-1.15\cdot10^{-3}$	$-1.97\cdot10^{-3}$	$3.89\cdot10^{-3}$
Silene ciliata	**$-2.56\cdot10^{-3}$**	$-4.31\cdot10^{-4}$	**0.23**	$-2.80\cdot10^{-3}$	$2.38\cdot10^{-2}$	$9.73\cdot10^{-3}$	$1.37\cdot10^{-3}$	$-1.04\cdot10^{-2}$	$8.51\cdot10^{-5}$

B)

lmer(trait$_i$~AltitudeQ+(1|pob))

	Individual size (cm^2)	Height (mm)	LT (µm)	SLA (mm^2·mg^{-1})	LDMC (mg·g^{-1})	LCC (mg·g^{-1})	δ^{13}C (‰)	LNC (mg·g^{-1})	δ^{15}N (‰)
	Estimate	Estimate	Estimate	Estimate	Estimate	Estimate	Estimate	Estimate	Estimate
Agrostis delicatula	$-4.24\cdot10^{-3}$	$-2.94\cdot10^{-2}$	-0.20	-0.10	2.69	$-4.82\cdot10^{-2}$	$3.23\cdot10^{-2}$	-0.14	**$-9.83\cdot10^{-2}$**
Armeria caespitosa	$3.02\cdot10^{-2}$	$9.97\cdot10^{-4}$	2.03	$-6.86\cdot10^{-2}$	1.70	-0.21	$2.14\cdot10^{-2}$	$1.25\cdot10^{-2}$	$3.27\cdot10^{-2}$
Deschampsia flexuosa	$8.65\cdot10^{-2}$	$-4.73\cdot10^{-3}$	-1.26	$8.63\cdot10^{-3}$	-0.48	-0.27	$-3.89\cdot10^{-2}$	$-6.91\cdot10^{-2}$	$-5.26\cdot10^{-2}$
Festuca curvifolia	$2.64\cdot10^{-2}$	$2.64\cdot10^{-4}$	5.22	$-1.14\cdot10^{-2}$	0.43	0.49	$1.36\cdot10^{-3}$	$-4.13\cdot10^{-2}$	0.10
Jasione crispa	$3.20\cdot10^{-2}$	**$-2.78\cdot10^{-2}$**	2.31	$2.93\cdot10^{-2}$	-1.15	-0.30	$6.37\cdot10^{-2}$	$4.92\cdot10^{-2}$	$3.51\cdot10^{-2}$
Juniperus communis subsp. *alpina*	$-2.15\cdot10^{-3}$	**$2.29\cdot10^{-2}$**	1.37	$-2.70\cdot10^{-2}$	0.78	0.46	$-1.91\cdot10^{-2}$	$-2.99\cdot10^{-2}$	$3.22\cdot10^{-2}$
Jurinea humilis	$-6.02\cdot10^{-3}$	$-1.25\cdot10^{-2}$	0.34	$8.99\cdot10^{-3}$	1.28	0.37	$8.01\cdot10^{-3}$	$8.94\cdot10^{-3}$	$-9.62\cdot10^{-2}$
Minuartia recurva	$5.37\cdot10^{-3}$	$4.41\cdot10^{-3}$	-0.47	0.10	0.76	0.20	$3.34\cdot10^{-2}$	-0.82	-1.91·10-01
Pilosella vahlii	$-8.32\cdot10^{-3}$	$-8.69\cdot10^{-3}$	**2.81**	$5.59\cdot10^{-2}$	-1.01	1.18	$1.66\cdot10^{-2}$	$-2.75\cdot10^{-2}$	$6.03\cdot10^{-2}$
Senecio carpetanus	$-4.25\cdot10^{-2}$	$-7.83\cdot10^{-3}$	0.56	$3.60\cdot10^{-2}$	-0.31	-0.12	$5.66\cdot10^{-2}$	0.22	0.22
Silene ciliata	$-1.39\cdot10^{-2}$	$-1.87\cdot10^{-2}$	**5.07**	$-6.08\cdot10^{-2}$	1.02	-0.19	$4.28\cdot10^{-2}$	-0.10	$-6.04\cdot10^{-3}$

C)

lmer(trait$_i$~Insolation+(1|pob))

	Individual size (cm^2)	Height (mm)	LT (µm)	SLA (mm^2·mg^{-1})	LDMC (mg·g^{-1})	LCC (mg·g^{-1})	δ^{13}C (‰)	LNC (mg·g^{-1})	δ^{15}N (‰)
	Estimate	Estimate	Estimate	Estimate	Estimate	Estimate	Estimate	Estimate	Estimate
Agrostis delicatula	0.90	-0.15	**192.02**	-3.63	-34.90	-6.22	-0.80	8.88	1.70
Armeria caespitosa	**5.36**	1.54	98.52	1.57	133.61	64.99	1.08	11.00	5.81
Deschampsia flexuosa	-3.96	-1.04	-66.58	$9.47\cdot10^{-2}$	22.00	-25.00	**-5.84**	-3.36	3.50
Festuca curvifolia	2.33	0.51	394.39	-2.87	149.55	**-62.64**	0.75	-5.04	3.48
Jasione crispa	1.52	-0.28	-36.31	5.69	12.40	17.02	2.75	2.70	2.31
Juniperus communis subsp. *alpina*	2.77	0.12	166.96	1.65	-119.92	-17.72	-0.97	11.22	7.64
Jurinea humilis	0.12	1.42	148.97	-2.90	65.64	-39.69	-3.00	-10.34	$-5.78\cdot10^{-2}$
Minuartia recurva	5.54	0.50	173.23	-9.02	-49.50	**50.42**	**4.23**	-23.86	5.35
Pilosella vahlii	-0.65	-0.95	-6.20	-1.71	74.77	-48.87	-0.94	6.00	3.39
Senecio carpetanus	-4.82	1.35	-57.61	-1.77	42.31	-57.53	-1.36	-35.23	2.32
Silene ciliata	-0.90	-1.07	333.43	-8.84	33.96	12.10	**4.66**	-6.17	0.76

D)

**$\mathrm{lmer}(\mathrm{trait}_i \sim \mathrm{SOC}+(1 \mid \mathrm{pob}))$**	Individual size (cm^2)	Height (mm)	LT (µm)	SLA ($mm^2 \cdot mg^{-1}$)	LDMC ($mg \cdot g^{-1}$)	LCC ($mg \cdot g^{-1}$)	$\delta^{13}C$ (‰)	LNC ($mg \cdot g^{-1}$)	$\delta^{15}N$ (‰)
	Estimate	Estimate	Estimate	Estimate	Estimate	Estimate	Estimate	Estimate	Estimate
Agrostis delicatula	0.12	$1.18 \cdot 10^{-2}$	2.67	0.22	**-9.71**	1.04	$-4.84 \cdot 10^{-2}$	$5.86 \cdot 10^{-2}$	0.21
Armeria caespitosa	$1.21 \cdot 10^{-2}$	$-5.44 \cdot 10^{-3}$	$9.82 \cdot 10^{-2}$	0.11	0.28	-1.81	**-0.17**	0.12	0.21
Deschampsia flexuosa	$-4.84 \cdot 10^{-2}$	**$-6.89 \cdot 10^{-2}$**	-6.79	$8.12 \cdot 10^{-2}$	0.57	-0.64	$9.65 \cdot 10^{-2}$	$-2.66 \cdot 10^{-3}$	**0.36**
Festuca curvifolia	$-6.17 \cdot 10^{-2}$	$-1.18 \cdot 10^{-2}$	-19.80	0.10	-5.28	-0.19	**-0.19**	$9.28 \cdot 10^{-2}$	0.28
Jasione crispa	0.10	$-4.69 \cdot 10^{-2}$	5.23	0.26	-3.03	1.62	$9.61 \cdot 10^{-2}$	0.66	0.15
Juniperus communis subsp. *alpina*	**0.22**	$5.31 \cdot 10^{-2}$	6.16	-0.20	6.69	2.03	$5.63 \cdot 10^{-3}$	0.18	**0.38**
Jurinea humilis	$2.95 \cdot 10^{-2}$	$3.63 \cdot 10^{-2}$	-4.47	$1.96 \cdot 10^{-2}$	0.57	-1.55	$5.85 \cdot 10^{-2}$	-0.39	0.30
Minuartia recurva	-0.16	**-0.13**	7.43	0.47	-6.27	-0.13	$-7.36 \cdot 10^{-2}$	1.45	0.40
Pilosella vahlii	$8.11 \cdot 10^{-2}$	$-2.46 \cdot 10^{-2}$	**-11.51**	-0.45	**7.89**	-0.86	$1.52 \cdot 10^{-2}$	0.56	**0.44**
Senecio carpetanus	$-7.62 \cdot 10^{-2}$	$-1.79 \cdot 10^{-2}$	13.41	-0.23	3.29	2.22	0.14	-0.27	0.16
Silene ciliata	0.11	$2.73 \cdot 10^{-2}$	-0.23	$-8.60 \cdot 10^{-2}$	2.66	1.27	$-5.09 \cdot 10^{-2}$	0.18	$-8.75 \cdot 10^{-2}$

E)

**$\mathrm{lmer}(\mathrm{trait}_i \sim \mathrm{NT}+(1 \mid \mathrm{pob}))$**	Individual size (cm^2)	Height (mm)	LT (µm)	SLA ($mm^2 \cdot mg^{-1}$)	LDMC ($mg \cdot g^{-1}$)	LCC ($mg \cdot g^{-1}$)	$\delta^{13}C$ (‰)	LNC ($mg \cdot g^{-1}$)	$\delta^{15}N$ (‰)
	Estimate	Estimate	Estimate	Estimate	Estimate	Estimate	Estimate	Estimate	Estimate
Agrostis delicatula	0.15	$2.00 \cdot 10^{-2}$	3.39	0.32	**-12.53**	1.40	$-8.60 \cdot 10^{-2}$	$5.42 \cdot 10^{-2}$	0.25
Armeria caespitosa	$3.07 \cdot 10^{-2}$	$8.68 \cdot 10^{-3}$	-1.73	0.14	0.18	-1.89	**-0.22**	$7.20 \cdot 10^{-2}$	0.25
Deschampsia flexuosa	$-7.24 \cdot 10^{-2}$	**$-8.53 \cdot 10^{-2}$**	-7.44	0.10	0.50	-0.95	0.12	$5.54 \cdot 10^{-3}$	**0.44**
Festuca curvifolia	$-8.36 \cdot 10^{-2}$	$-1.41 \cdot 10^{-2}$	-25.30	0.15	-7.82	-0.69	-0.22	0.12	0.35
Jasione crispa	0.11	$-3.60 \cdot 10^{-2}$	1.53	0.57	-3.50	2.57	$9.45 \cdot 10^{-2}$	0.75	0.16
Juniperus communis subsp. *alpina*	**0.28**	$6.30 \cdot 10^{-2}$	7.60	-0.25	8.35	2.60	$2.99 \cdot 10^{-3}$	0.18	**0.49**
Jurinea humilis	$9.60 \cdot 10^{-2}$	$9.39 \cdot 10^{-2}$	-5.89	$2.69 \cdot 10^{-2}$	1.23	-2.24	$5.01 \cdot 10^{-2}$	-0.32	**0.45**
Minuartia recurva	-0.21	-0.16	7.85	0.42	-2.14	-0.27	-0.14	1.74	0.37
Pilosella vahlii	0.10	$-3.35 \cdot 10^{-2}$	**-14.83**	-0.49	8.78	-3.41	$3.51 \cdot 10^{-3}$	0.68	**0.54**
Senecio carpetanus	-0.14	$1.42 \cdot 10^{-2}$	13.81	-0.25	2.63	0.18	$5.94 \cdot 10^{-2}$	-1.46	$6.81 \cdot 10^{-2}$
Silene ciliata	0.14	$1.79 \cdot 10^{-2}$	-0.16	$-5.16 \cdot 10^{-2}$	0.36	1.67	$-4.16 \cdot 10^{-2}$	0.44	-0.26

F)

$lmer(trait_i$~Temperature+(1|pob)	Individual size (cm^2)	Height (mm)	LT (µm)	SLA ($mm^2 \cdot mg^{-1}$)	LDMC ($mg \cdot g^{-1}$)	LCC ($mg \cdot g^{-1}$)	$\delta^{13}C$ (‰)	LNC ($mg \cdot g^{-1}$)	$\delta^{15}N$ (‰)
	Estimate	Estimate	Estimate	Estimate	Estimate	Estimate	Estimate	Estimate	Estimate
Agrostis delicatula	0.33	0.17	-6.95	-1.16	14.52	-1.71	0.75	-1.17	0.42
Armeria caespitosa	-0.25	-0.18	1.19	-0.32	7.00	-8.33	0.32	**-2.06**	-0.69
Deschampsia flexuosa	0.24	0.11	-34.31	$6.04 \cdot 10^{-2}$	3.22	-4.13	0.19	**-2.25**	-0.34
Festuca curvifolia	$-8.28 \cdot 10^{-2}$	$-1.07 \cdot 10^{-2}$	-35.61	0.21	-9.33	6.97	$4.54 \cdot 10^{-2}$	-1.49	0.12
Jasione crispa	-0.13	$2.81 \cdot 10^{-2}$	0.91	-0.49	-2.92	**-8.84**	-0.66	-1.44	-0.86
Juniperus communis subsp. *alpina*	0.21	0.19	-24.26	-0.37	20.55	8.34	-0.17	**-1.10**	-0.50
Jurinea humilis	-0.27	-0.19	-13.01	0.24	-11.93	2.48	0.45	**-2.79**	-0.94
Minuartia recurva	$-2.92 \cdot 10^{-2}$	$9.60 \cdot 10^{-2}$	-8.98	0.41	-5.52	-1.29	$7.14 \cdot 10^{-2}$	-1.13	-0.45
Pilosella vahlii	-0.13	$5.90 \cdot 10^{-2}$	-14.90	-0.73	7.58	17.12	0.22	-1.81	-0.11
Senecio carpetanus	0.39	-0.15	-10.68	0.68	-13.38	-2.26	0.34	2.64	0.85
Silene ciliata	0.23	0.11	-18.04	0.43	-7.74	-4.14	$-9.73 \cdot 10^{-2}$	-1.03	-0.47

G)

$lmer(trait_i$~Moisture+(1|pob)	Individual size (cm^2)	Height (mm)	LT (µm)	SLA ($mm^2 \cdot mg^{-1}$)	LDMC ($mg \cdot g^{-1}$)	LCC ($mg \cdot g^{-1}$)	$\delta^{13}C$ (‰)	LNC ($mg \cdot g^{-1}$)	$\delta^{15}N$ (‰)
	Estimate	Estimate	Estimate	Estimate	Estimate	Estimate	Estimate	Estimate	Estimate
Agrostis delicatula	$-1.52 \cdot 10^{-2}$	$-2.49 \cdot 10^{-3}$	0.11	$4.50 \cdot 10^{-2}$	-0.46	0.13	**$-3.69 \cdot 10^{-2}$**	$5.30 \cdot 10^{-2}$	$-1.21 \cdot 10^{-2}$
Armeria caespitosa	$-2.05 \cdot 10^{-3}$	$5.23 \cdot 10^{-3}$	-0.38	$3.54 \cdot 10^{-2}$	-1.17	0.16	$-1.47 \cdot 10^{-2}$	$6.28 \cdot 10^{-2}$	$2.87 \cdot 10^{-2}$
Deschampsia flexuosa	$-1.46 \cdot 10^{-2}$	$-1.10 \cdot 10^{-3}$	1.95	$-6.37 \cdot 10^{-3}$	$-1.25 \cdot 10^{-3}$	0.19	$-8.79 \cdot 10^{-3}$	**0.10**	$1.05 \cdot 10^{-2}$
Festuca curvifolia	$-4.54 \cdot 10^{-4}$	$5.24 \cdot 10^{-3}$	1.37	$-9.84 \cdot 10^{-3}$	0.60	-0.21	$1.00 \cdot 10^{-2}$	$8.18 \cdot 10^{-3}$	$-1.11 \cdot 10^{-3}$
Jasione crispa	$-3.17 \cdot 10^{-3}$	$-5.01 \cdot 10^{-3}$	-0.34	$7.86 \cdot 10^{-2}$	-0.45	0.30	$2.21 \cdot 10^{-2}$	$5.05 \cdot 10^{-2}$	$4.71 \cdot 10^{-2}$
Juniperus communis subsp. *alpina*	$-5.77 \cdot 10^{-3}$	$-4.31 \cdot 10^{-3}$	1.53	$-7.26 \cdot 10^{-3}$	$1.77 \cdot 10^{-2}$	-0.15	$4.77 \cdot 10^{-3}$	$3.64 \cdot 10^{-2}$	$2.96 \cdot 10^{-2}$
Jurinea humilis	$-4.02 \cdot 10^{-4}$	$7.26 \cdot 10^{-3}$	0.87	$-2.03 \cdot 10^{-2}$	0.34	$-5.90 \cdot 10^{-2}$	$-2.72 \cdot 10^{-2}$	$9.97 \cdot 10^{-2}$	$6.00 \cdot 10^{-2}$
Minuartia recurva	$-1.66 \cdot 10^{-2}$	$-8.32 \cdot 10^{-3}$	-0.22	$7.32 \cdot 10^{-3}$	1.30	$-2.45 \cdot 10^{-2}$	$-1.78 \cdot 10^{-2}$	$-6.34 \cdot 10^{-2}$	$-4.71 \cdot 10^{-2}$
Pilosella vahlii	$8.10 \cdot 10^{-3}$	$4.61 \cdot 10^{-3}$	0.59	$8.84 \cdot 10^{-2}$	-0.94	-0.11	$-8.41 \cdot 10^{-3}$	$9.79 \cdot 10^{-2}$	$-2.15 \cdot 10^{-3}$
Senecio carpetanus	**$-3.08 \cdot 10^{-2}$**	$4.54 \cdot 10^{-3}$	2.07	$-4.96 \cdot 10^{-3}$	-0.16	-0.12	$-1.52 \cdot 10^{-2}$	$-8.53 \cdot 10^{-2}$	$1.55 \cdot 10^{-2}$
Silene ciliata	$-1.86 \cdot 10^{-2}$	$-4.00 \cdot 10^{-3}$	1.77	$-2.71 \cdot 10^{-3}$	0.15	$5.64 \cdot 10^{-2}$	$1.29 \cdot 10^{-4}$	$-1.63 \cdot 10^{-2}$	$1.41 \cdot 10^{-2}$
