# Supplementary material for: Plant Trait Variation along an Altitudinal Gradient in Mediterranean High Mountain Grasslands: Controlling the Species Turnover Effect
Source: PLoS One. 2015 Mar 16;10(3):e0118876. doi: 10.1371/journal.pone.0118876 (PMC4361585; doi:10.1371/journal.pone.0118876)
Supplement: S3 Table — (PDF) [file pone.0118876.s005.pdf]

S3 Table. Species turnover and intra-specific trait variability relative contribution (in %) on CWM and FD response to altitude. Three ANOVAs and Sum of squared (SS) decomposition for **A)** CWM_s (species turnover and intra-specific effects), CWM_f (species turnover effect) and differences between them (intra-specific trait variability effect) and **B)** FD_s (species turnover and intra-specific effects), FD_f (species turnover effect) and their differences (intra-specific trait variability effect); to establish the relative effect on CWM and FD response to altitude respectively of species turnover and intra-specific trait variability for each functional trait considered. Covariation effect consider the positive (positive covariation term) or negative (negative covariation term) correlation between species turnover and intra-specific effects. Significant values ($p < 0.05$) are marked in bold.

A) CWM

Functional trait	Turnover effect	Intra-specific effect	Covariance effects	Total effect
Individual size (cm^2)	3,1	0,0	-0,4	2,7
Height (mm)	3,9	5,2	-9,0	0,1
LT (µm)	13,3	16,3	-29,4	0,2
SLA ($mm^2 \cdot mg^{-1}$)	50,2	2,2	-20,9	31,5
LDMC ($mg \cdot g^{-1}$)	21,6	18,1	-39,5	0,2
LCC ($mg \cdot g^{-1}$)	7,8	2,9	9,5	20,2
$\delta^{13}C$ (‰)	14,3	**86,4**	-70,3	30,4
LNC ($mg \cdot g^{-1}$)	3,2	0,0	0,8	4,0
$\delta^{15}N$ (‰)	0,2	24,0	4,0	28,1

B) FD (Rao)

Functional trait	Turnover effect	Intra-specific effect	Covariation effect	Total effect
Individual size (cm^2)	5.9	3.6	-9.3	0.3
Height (mm)	6.0	9.6	-15.2	0.4
LT (µm)	25.5	2.1	14.7	42.2
SLA ($mm^2 \cdot mg^{-1}$)	**65.1**	1.1	17.1	**83.4**
LDMC ($mg \cdot g^{-1}$)	27.7	7.5	28.8	**63.9**
LCC ($mg \cdot g^{-1}$)	62.9	10.4	-51.1	22.1
$\delta^{13}C$ (‰)	19.9	**18.0**	37.9	**75.8**
LNC ($mg \cdot g^{-1}$)	7.6	3.7	-10.6	0.7
$\delta^{15}N$ (‰)	4.6	**29.8**	23.5	**57.9**
